# Time Trends in Major Adverse Cardiovascular Events After Percutaneous Coronary Intervention: Meta-Analysis on Sex Differences

**DOI:** 10.1016/j.jacadv.2024.101526

**Published:** 2025-01-08

**Authors:** Shayan Shojaei, Asma Mousavi, Hamidreza Soleimani, Fatemeh Takaloo, Peyvand Parhizkar Roudsari, Dorsa Salabat, Amirreza Shahmohamady, Mehdi Mehrani, Parisa Seilani, Haleh Ashraf, John Nelson, Rosy Thachil, Fady Iskander, Safi U. Khan, Nasir Khurram, Kaveh Hosseini

**Affiliations:** a Cardiac Primary Prevention Research Center, Cardiovascular Diseases Research Institute, Tehran University of Medical Sciences, Tehran, Iran; b Tehran Heart Center, Cardiovascular Diseases Research Institute, Tehran University of Medical Sciences, Tehran, Iran; c California Cardiovascular Institute, California, USA; d Elmhurst Hospital Center/Mount Sinai School of Medicine, New York, USA; e MedStar Union Memorial Hospital, Maryland, USA; f Internal Medicine Department, West Virginia University Hospitals, West Virginia, USA; g DeBakey Heart and Vascular Center, Houston Methodist Hospital, Houston, USA

**Keywords:** acute coronary syndrome, major adverse cardiovascular events, percutaneous coronary intervention, sex differences

## Abstract

**Background:**

Percutaneous coronary intervention (PCI) is considered the procedure of choice for patients with acute coronary syndrome (ACS), as it significantly improves cardiovascular outcomes. However, considerable uncertainty persists regarding the potential sex differences in PCI outcomes, due to conflicting results in previous studies.

**Objectives:**

This meta-analysis aims to evaluate potential sex-related differences in cardiovascular adverse outcomes after PCI among ACS patients.

**Methods:**

The primary outcome was major adverse cardiovascular events (MACE) and its components. Outcomes were examined in various time frames including: short-term (within 1 month after PCI), mid-term (within 1 year), and long-term (within >1 year). A random effects model was used to estimate risk ratios (RR) and 95% CIs.

**Results:**

Among 32 trials, at short-term, PCI was associated with a higher risk of MACE (risk ratio [RR]: 1.43; 95% CI: 1.10-1.86), all-cause mortality (RR: 2.51; 95% CI: 1.70-3.71), and myocardial infarction (RR: 1.33; 95% CI: 1.00-1.77) in women compared with men. Over the long-term, women had a higher risk of MACE (RR: 1.11; 95% CI: 1.01-1.22), all-cause mortality (RR: 1.29; 95% CI: 1.17-1.42), and cardiovascular mortality (RR: 1.30; 95% CI: 1.11-1.52), when compared with men. However, the analysis for stroke and repeat revascularization showed no significant difference between the 2 groups in the long- and short-term.

**Conclusions:**

In the meta-analysis of PCI-related trials in ACS, women have a higher risk of adverse cardiovascular outcomes compared with men.

Cardiovascular disease is recognized as the leading cause of death worldwide and one of the most significant health challenges.^1^^,^^2^ Several studies have highlighted the differences in the incidence of major adverse cardiovascular events (MACE) between men and women.^3^ Although, in some studies, women exhibit lower age-adjusted incidence, prevalence, and mortality from cardiovascular diseases compared with men,^3^ women have a higher risk profile for cardiovascular problems like diabetes and chronic kidney disease in comparison to men^4^^,^^5^ and increased adjusted risk of both in-hospital and 30-day mortality rates after ST-segment elevation myocardial infarction (STEMI).^6^^,^^7^

Recent clinical trials have suggested that women may have experienced worse outcomes, specifically primary bleeding, compared with men after percutaneous coronary intervention (PCI).^8^ However, these findings should be interpreted with caution due to the limitations of the study designs. Most of the articles related to this subject are often conducted as substudy analyses. Due to the design, analyses are underpowered which lead to uncertainty about any potential sex-related differences.^9^

Since limited data exist regarding sex-related differences in MACE after PCI for acute coronary syndrome (ACS) in clinical trials, we conducted a systematic review and meta-analysis focusing on randomized clinical trials that reported sex-specific data or its post hoc analyses evaluating these outcomes.

## Method

### Protocol

The present study was reported following the Preferred Reporting Items for Systematic Reviews and Meta-Analyses (PRISMA) guidelines and conducted as per Cochrane guidelines,^10^ and the study protocol was registered in the International Prospective Register for Systematic Reviews (PROSPERO) with the registration code of CRD42024505637.

### Search strategy and study selection

To identify relevant studies, we conducted a literature review using PubMed, Embase, Web of Science, and the Cochrane clinical trial databases from inception to January 2024. No language restrictions were applied. We screened reference lists of eligible studies and relevant reviews. The detailed search strategies are provided in Supplemental Table 1.

Our inclusion criteria were randomized clinical trials and subgroup analyses of trials that reported MACE after PCI in patients with ACS based on sex. We excluded studies based on these criteria: not reporting the baseline characteristics of the patients, studies with emergency coronary artery bypass graft, studies including patients presenting with cardiogenic shock, conference abstracts, animal studies, non-English publications, and those without full text available.

Two authors (A.M. and S.S.) screened the titles and abstracts of all the studies independently to determine the ones qualified for full-text assessment and discrepancies were resolved through discussion with a senior author (K.H.).

### Outcomes of interest

The primary outcomes of our study were MACE and its individual components (all-cause mortality, cardiovascular mortality, MI, stroke, and repeat revascularization), while secondary outcomes were stent thrombosis and major bleeding. Outcomes were evaluated in short-term (within 1 month after PCI), mid-term (within 1 year), and long-term (within >1 year).

### Risk of bias assessment

Risk of Bias 2 instructions were used to assess the risk of bias in the included studies.^11^ Two authors (D.S. and S.S.) independently assessed all the studies, and the discrepancies were resolved by consulting with a senior author (K.H.).

### Data extraction

Two authors (A.M. and S.S.) independently assessed the included studies and extracted relevant data including author, publication year, sample sizes, mean age, sex, mean body mass index, background disease, cardiovascular risk factors (such as diabetes, smoking, dyslipidemia, and hypertension status), type of stent, lab data, angiographic features, and outcomes.

### Statistical analysis

We used R programming language (R for Windows, Version 4.1.3) and RStudio, Version 1.1.463 (Posit PBC) and the “tidyverse” and “meta” statistical packages to perform the meta-analysis in our study. We estimated relative risks (RRs) with 95% CIs. We determined between-study statistical heterogeneity via I2 parameters with I2 >50% indicating significant heterogeneity. We used a random effect model to estimate the effect size of the pooled data. Sensitivity analyses—leave one out and fixed effect analysis—were performed to explore heterogeneity sources. The funnel plots were generated for variables reported in more than 10 studies.

## Results

**Figure 1:**
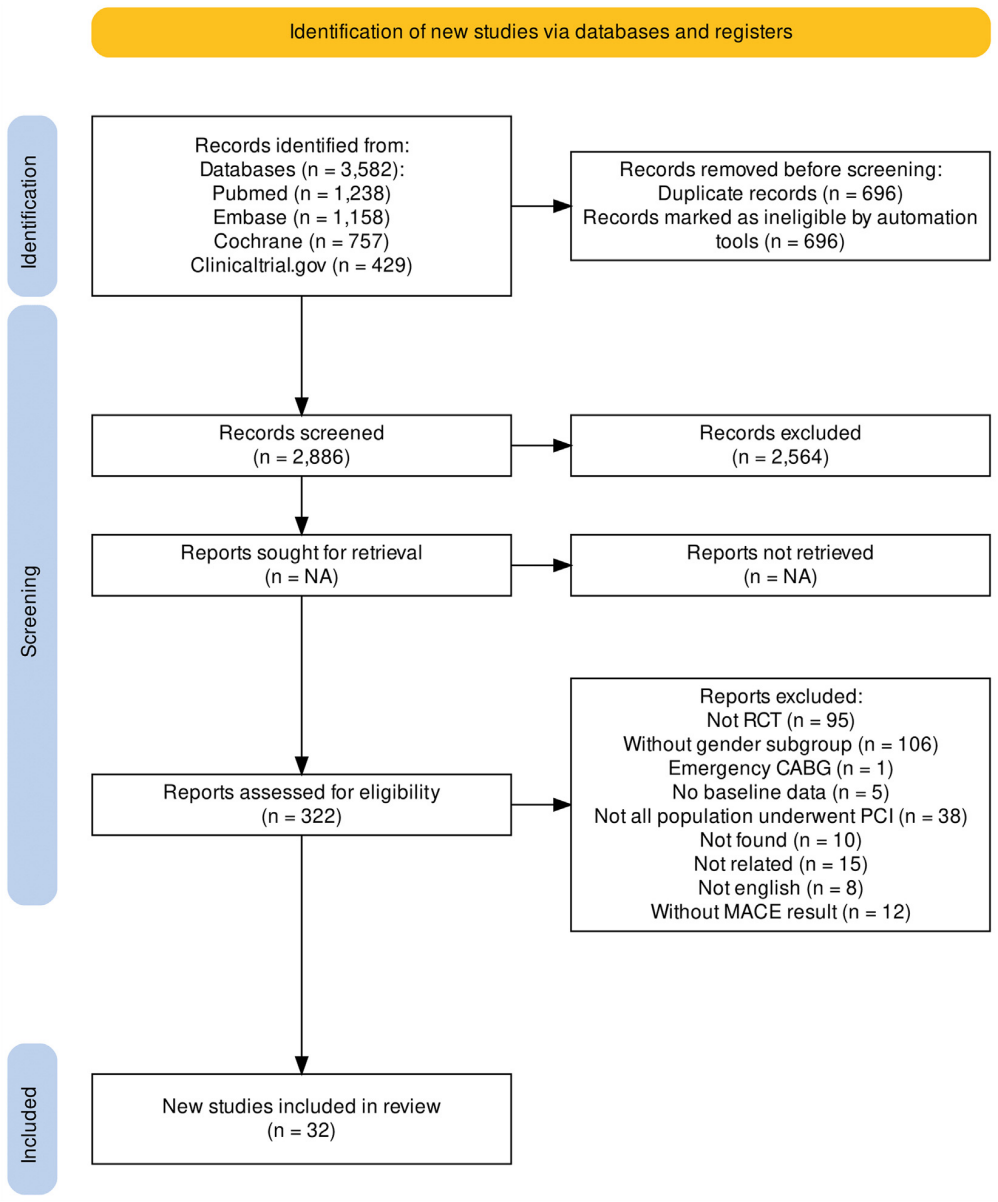
Preferred Reporting Items for Systematic Reviews and Meta-analyses Charts CABG = coronary artery bypass graft; MACE = major adverse cardiovascular events; RCT = randomized clinical trial.

A comprehensive search yielded a total of 3,582 studies. After removing duplicates and screening the title and abstract, 32 trials^8^^,^^9^^,^^12^, ^13^, ^14^, ^15^, ^16^, ^17^, ^18^, ^19^, ^20^, ^21^, ^22^, ^23^, ^24^, ^25^, ^26^, ^27^, ^28^, ^29^, ^30^, ^31^, ^32^, ^33^, ^34^, ^35^, ^36^, ^37^, ^38^, ^39^, ^40^, ^41^ remained for our final analysis. The inter-readers’ agreement was high (Kappa coefficient 90%). Figure 1 depicts the study selection process according to the PRISMA guidelines.

The total number of individuals across the 32 articles was 78,846. Among them, 59,491 (75.5%) participants were men, while 19,355 (24.5%) were women. The mean age of participants was 63.98 (range: 56.7-78.8) years, and the mean body mass index was 27.97 (range: 22.9-31.3) kg/m^2^. The left ventricular ejection fraction varied between 45.8 and 61.3. Hypertension was more frequently encountered in women (68.2%) as opposed to men (57.4%). Moreover, the prevalence of diabetes was observed to be 30.7% in men and 29.5% in women. Furthermore, the smoking rates among patients were 24.5% for women and 37.3% for men. The median duration of follow-up was 14 months (Q1, Q3: 12, 36). Table 1 provides an overview of the baseline characteristics in both groups across the included studies.

**Table 1:** Baseline Characteristics of Included Studies

Study Characteristics			Male Group							Female Group						
First Author/Year	Country	Follow-Up (Days)	N	Mean Age (y)	Mean BMI (kg/m^2^)	HTN (%)	DM (%)	Dyslipidemia (%)	Smoker (%)	N	Mean Age (y)	Mean BMI (kg/m^2^)	HTN (%)	DM (%)	Dyslipidemia (%)	Smoker (%)
Berry et al/2018^12^	USA	365	8,723	60.7	30.2	71.5	27.3	N/A	27.9	2,925	63.3	31.1	79.1	35	N/A	25.9
Chako et al/2006^13^	Multinational	365	4,465	N/A	29.5	63.8	25.6	N/A	27.6	1,537	N/A	30.1	76	31.6	N/A	23.7
Eccleston et al/2021^14^	Multinational	420	2,664	69.4	N/A	N/A	35.6	N/A	N/A	825	72.52	N/A	N/A	42.0	N/A	N/A
Ferrante et al/2012^15^	Multinational	1,080	565	61.5	N/A	54.7	14.1	37.8	40.3	179	70.5	N/A	66.7	15.6	41.05	28.7
Gabani et al/2022^9^	Multinational	3,650	1,244	59.8	27.4	45.6	17	42.9	79.5	254	67.93	27 ± 5	62.2	18.1	47.6	36.6
Gargiulo et al/2016^16^	Italy	730	1,511	67.8	26.8	69.2	23.9	54.55	25.5	459	73.5	26.2	80.4	25.0	55.1	18.2
Gimenez et al/2023^31^	Multinational	1,080	557	67	28.2	85.4	32.2	69.2	22	201	70	28.5	90.5	36.8	69.8	17.4
Guagliumi et al/2014^18^	Italy	365	70	65.3	26.1	58.6	8.6	30	55.7	70	67.8	25.5	55.7	15.7	27.1	51.4
Hansen et al/2013^19^	Multinational	730	1,749	62	N/A	60.5	15.5	64	34.5	565	68.5	N/A	69	16	63	29.5
Hara et al/2020^20^	Multinational	1,825	690	64.3	28	67.3	23.9	78.8	20	213	68.3	28.3	74.2	30.1	78.2	13.6
Gross et al/2019^17^	Multinational	365	2,052	58.1	28.3	59.4	N/A	41.35	45	558	61.1	28.15	68.2	N/A	40.5	46.4
Kim et al/2012^21^	Multinational	730	744	63.1	28.05	60.7	24.1	71	30.7	261	68.35	28.2	72.1	26.3	76.5	25.2
Lansky et al/2009^22^	USA	1,825	687	61.7	30.24	72.3	25	72.6	24.1	314	65.7	31.24	80.9	36.4	73.1	20.7
Lansky et al/2005^23^	USA	365	1,520	57.0	N/A	29	14	35.9	45.3	562	66.0	N/A	59.3	25.7	43.2	37.4
Lee et al/2023^24^	South Korea	360	2,428	59.4	25.05	48.0	25.2	59.55	45.1	628	67	24.45	59.7	35.5	63.85	7.45
Madan et al/2022^25^	Multinational	360	3,983	61	28	60.1	25.2	52.1	26.4	1,293	67	27.7	71.2	33.1	52.8	18.2
Mehilli et al/2012^27^	Germany	360	1,322	66.4	27.85	84.9	28.4	68.2	25.6	399	71.15	27.6	87.2	31.0	69.65	17.8
Mehilli et al/2007^26^	Germany	30	1,524	64.9	27.35	59.4	24.1	60.2	23.9	498	70.4	26.8	74.9	33.8	62.95	17
Mehran et al/2020^28^	Multinational	720	1,694	74.5	27.37	76.4	33.1	61.65	13.1	738	78.29	27.26	84.2	33.5	63.95	6.85
Meller et al/2013^29^	Multinational	360	935	57	N/A	43.4	14.9	36.5	47.1	366	67	N/A	59.8	25.4	45.4	36.1
Regueiro et al/2014^30^	USA	720	1,244	59.8	27.4	45.6	17.0	42.9	79.5	254	68	26.95	61.8	18.3	47.55	36.1
Serruys et al/2018^32^	Multinational	1,080	722	65.8	N/A	72.2	28.7	70.2	N/A	226	66.8	N/A	81	32.7	73.5	N/A
Shin et al/2023^34^	Korea	1,560	2,198	62.6	24.65	58.1	35.4	43.3	34.1	795	69.7	24.35	70.8	43.2	51.05	5.55
Shin et al/2018^33^	Japan	365	537	56.9	24.6	41.1	25.2	43.55	65	163	69.35	23.15	61.7	18.3	41.45	11.4
Sia et al/2015^35^	Finland	720	629	61.6	N/A	46.3	15.1	46.1	36.1	198	67.4	N/A	61.6	22.7	49.5	25.8
Tandjung et al/2013^36^	Netherlands	365	1,009	62.9	27.7	52.5	19.8	59	25.4	382	67.55	27.8	63.6	26.4	59.9	21.7
Tomey et al/2014^37^	USA	30	334	58	26.8	27.2	9.9	13.8	47.7	118	66.5	25.7	43.2	15.3	21.2	41.4
Tsujita et al/2010^38^	USA	365	2,441	N/A	26.6	44.6	19.2	44.6	41.6	712	N/A	26.25	48.0	22.9	48.05	35.4
Venetsanos/2019^39^	Sweden	180	4,406	65.8	27.6	49.6	16.2	32.05	58.9	1,592	70.95	27.15	57.7	17.9	29.8	55
Verdoia et al/2021^40^	Netherlands	720	1,195	59.9	26.95	46.8	19.5	45.2	45	300	64.35	27.25	64.4	25.2	47	31.9
Vogel et al/2021^8^	Multinational	360	5,421	63.3	28.5	71.1	36.9	60.9	23.2	1,698	65.55	28.8	76.5	36.4	58.9	17
Yang et al/2022^41^	Korea	3,600	228	61.9	24.5	53.5	31.6	42.5	38.6	72	61.3	24.8	56.9	41.7	41.7	1.4

BMI = body mass index; DM = diabetes mellitus; HTN = hypertension.

Supplemental Tables 2 and 3 present angiographic details, including stent type, infarct artery, number of stents per person, preintervention/postintervention thrombosis in myocardial infarction flow, PCI approach, and history of PCI, coronary artery bypass graft, and MI in participants.

### Quality assessment

In this study, the Risk of Bias 2 tool was used to assess the quality of the 32 included studies. Overall, 22 out of the 32 studies were rated as high quality, while the remaining 10 studies were rated as moderated quality. Supplemental Table 4 summarizes the quality assessment of the included studies.

## Primary outcomes

### Short-term outcomes

**Figure 2:**
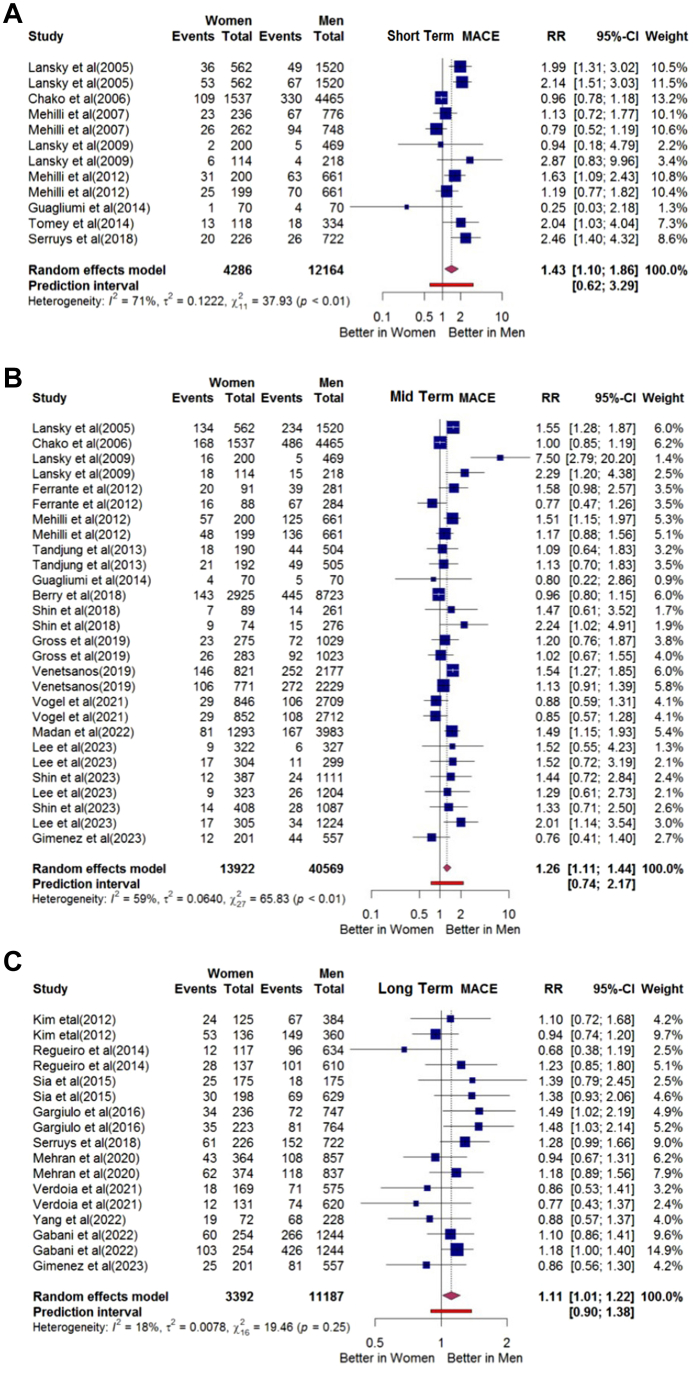
Sex Differences in Major Adverse Cardiovascular Events Across Various Time Frames (A) Risk ratios of short-term major adverse cardiovascular events. (B) Risk ratios of mid-term major adverse cardiovascular events. (C) Risk ratios of long-term major adverse cardiovascular events. Abbreviation as in Figure 1.

A trend was observed regarding higher risk of MACE, all-cause mortality, and MI in women than men after PCI (risk ratio [RR]: 1.43; 95% CI: 1.10-1.86; I^2^: 71%), (RR: 2.51; 95% CI: 1.70-3.71; I^2^: 36%), and (RR: 1.33; 95% CI: 1.00-1.77; I^2^: 53%) respectively (Figure 2A, Supplemental Figures 1A and 1B). There was no significant difference in cardiovascular mortality, stroke, and repeat revascularization between the men and women (RR: 2.16; 95% CI: 0.80-5.85; I^2^:0%; RR: 4.75; 95% CI: 0.83-27.31; I^2^: 20%; and (RR: 1.16; 95% CI: 0.89-1.51; I^2^: 0%, respectively) (Supplemental Figures 1C to 1E).

### Mid-term outcomes

Analysis of mid-term outcomes revealed that women may experience a significantly higher risk of MACE, all-cause mortality, cardiovascular mortality, MI, and stroke in women than in men (RR: 1.26; 95% CI: 1.11-1.44; I^2^: 59%); RR: 1.44; 95% CI: 1.21-1.72; I^2^: 54%; RR: 1.29; 95% CI: 1.00-1.66; I^2^: 38%; RR: 1.17; 95% CI: 1.06-1.29; I^2^: 0%; and RR: 1.47; 95% CI: 1.13-1.92; I^2^: 0%, respectively). (Figure 2B, Supplemental Figures 2A to 2D). There was no significant difference in repeat revascularization between both groups after PCI (RR: 1.13, 95% CI: 0.94-1.35; I^2^: 27%) (Supplemental Figure 2D).

### Long-term outcomes

**Figure 3:**
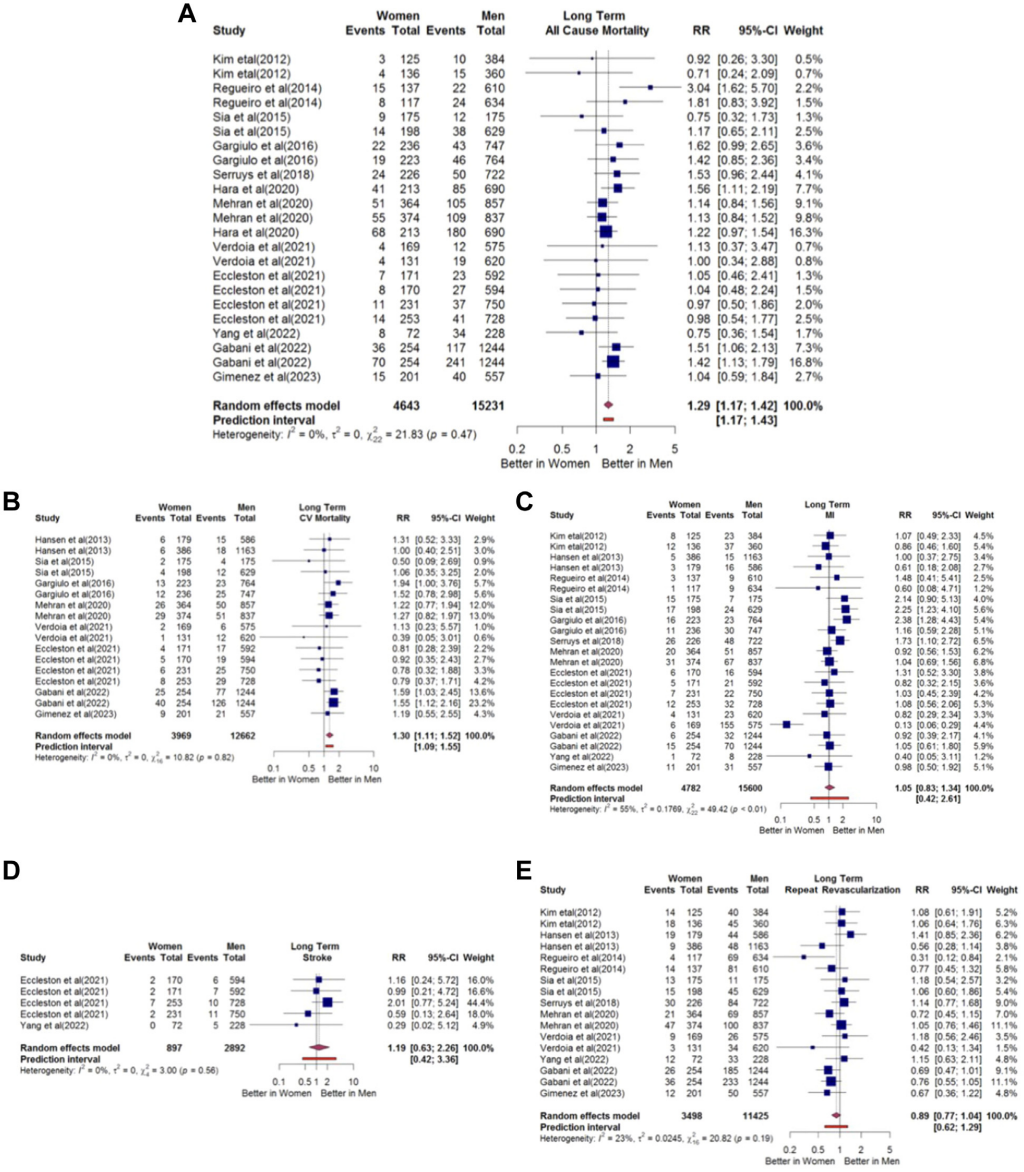
Sex Differences in All Cause Mortality, Cardiovascular Mortality, Myocardial Infarction, Stroke, and Repeat Revascularization Over the Long Term (A) Risk ratio of long-term all-cause mortality. (B) Risk ratio of long-term cardiovascular mortality. (C) Risk ratio of long-term myocardial infarction. (D) Risk ratio of long-term stroke mortality. (E) Risk ratio of long-term repeat revascularization. CV = cardiovascular; MI = myocardial infarction.

In long-term outcomes, women had a higher risk of MACE, all-cause mortality, and cardiovascular mortality compared with men (RR: 1.11; 95% CI: 1.01-1.22; I^2^: 18%; RR: 1.29; 95% CI: 1.17-1.42; I^2^: 0%; and RR: 1.30; 95% CI: 1.11-1.52; I^2^: 0%, respectively) (Figures 2C, 3A, and 3B). The analysis for MI, stroke, and repeat revascularization showed no significant difference between sexes (RR: 1.05; 95% CI: 0.83-1.34; I^2^: 55%; RR: 1.19; 95% CI: 0.63-2.26; I^2^: 0%; and RR: 0.89; 95% CI: 0.77-1.04; I^2^: 23%, respectively) (Figures 3C to 3E).

The detailed results of the primary outcomes for different periods of time are summarized in Table 2.

**Table 2:** Primary Outcomes (MACE) in Short-Term, Mid-Term, and Long-Term Duration of Follow-Up

Duration of Follow-Up	Number of Trials	RR (95% CI)	I^2^,%	*P* Value for Heterogeneity
MACE				
Short-term	8	1.43 (1.10-1.86)	71%	<0.01
Mid-term	16	1.26 (1.11-1.44)	59%	<0.01
Long-term	10	1.11 (1.01-1.22)	18%	0.25
All-cause mortality				
Short-term	8	2.51 (1.70-3.71)	36%	0.11
Mid-term	17	1.44 (1.21-1.72)	54%	<0.01
Long-term	12	1.29 (1.17-1.42)	0%	0.47
CV mortality				
Short-term	3	2.16 (0.80-5.85)	0%	0.56
Mid-term	13	1.29 (1.00-1.66)	38%	0.03
Long-term	8	1.30 (1.11-1.52)	0%	0.82
MI				
Short-term	8	1.33 (1.00-1.77)	53%	0.01
Mid-term	16	1.17 (1.06-1.29)	0%	0.53
Long-term	12	1.05 (0.83-1.34)	55%	<0.01
Stroke				
Short-term	3	4.75 (0.83-27.31)	20%	0.29
Mid-term	9	1.47 (1.13-1.92)	0%	0.67
Long-term	2	1.19 (0.63-2.26)	0%	0.56
Repeat revascularization				
Short-term	8	1.16 (0.89-1.51)	0%	0.63
Mid-term	12	1.13 (0.94-1.35)	27%	0.12
Long-term	10	0.89 (0.77-1.04)	23%	0.19

CV = cardiovascular; MACE = major adverse cardiovascular events; MI = myocardial infarction.

### Secondary outcomes

Rates of stent thrombosis in short-, mid-, and long-term analyses were not different between sexes (RR: 1.52; 95% CI: 0.82-2.82; I^2^: 0%; RR: 1.00; 95% CI: 0.76-1.32; I^2^: 0%; and (RR: 0.86; 95% CI: 0.55-1.34; I^2^: 0%, respectively) (Supplemental Figure 3). Major bleeding was seemed to be higher in women vs men in short- and mid-term analyses (RR: 3.05; 95% CI: 1.81-5.15; I^2^: 27%) and (RR: 1.88; 95% CI: 1.63-2.17; I^2^: 9%), but there was no significant difference between 2 groups in the long-term analysis (RR: 1.12; 95% CI: 0.89-1.41; I^2^: 0%) (Supplemental Figure 4). There was no significant heterogeneity observed in any of the analyses.

The detailed results of the secondary outcomes for different durations are provided in Supplemental Table 5.

### Sensitivity analysis

Due to the high heterogeneity among the articles, we decided to use random effect analysis for our study (Supplemental Table 6). We utilize the leave-one-out method to investigate any source of heterogeneity. However, there was not any impactful source to resolve. Egger’s test and funnel plots were used to assess publication bias in each of the previously mentioned outcomes across different time frames. Overall, the risk of publication bias was considered to be low (Supplemental Figures 5A to 5, 6A to 6E, 7A to 7D, 8A and 8B, 9A and 9B, and 10A to 10C).

### Meta-regression

A meta-regression analysis examining the relationship between the risk of MACE and one-unit increase in each baseline characteristic variable revealed both positive and negative associations, but none were statistically significant. In contrast, an analysis of baseline characteristics in the female group indicated a significant decrease in the risk of all-cause mortality with each one-unit increase in left ventiruclar ejection fraction, shown by a *P* value of 0.03 and a strong correlation with a beta of −0.1. Other variables, however, did not show statistically significant associations.

## Discussion

The findings of this meta-analysis reveal significant differences in cardiovascular outcomes and mortality between women and men after PCI among ACS patients. Women exhibited higher rates of all-cause mortality in all 3 time frames as well as for each of the other primary outcomes: MI in short- and mid-term, cardiovascular mortality in mid- and long-term, and stroke in mid-term. Major bleeding in short- and mid-term was also higher in women compared to men. However, repeat revascularization and stent thrombosis showed no significant difference between the 2 groups (Central Illustration).

**Central Illustration:**
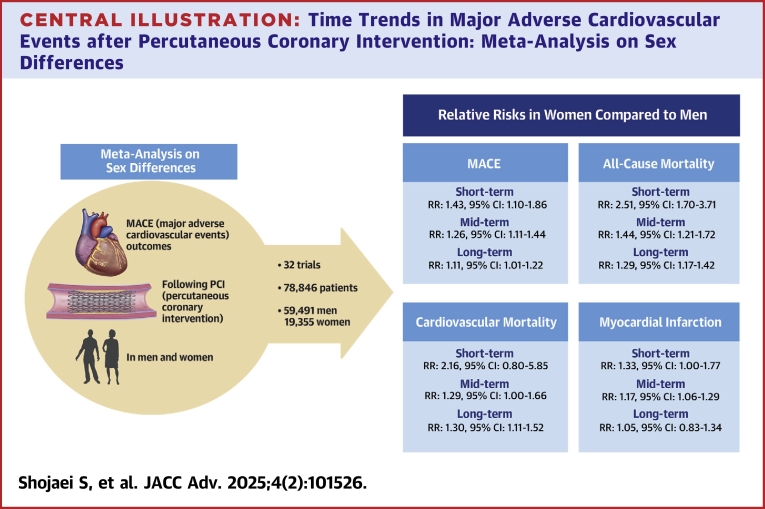
Time Trends in Major Adverse Cardiovascular Events after Percutaneous Coronary Intervention: Meta-Analysis on Sex Differences

Current meta-analysis indicates a greater incidence of cardiovascular events and mortality among women following PCI for ACS patients. Similar findings have been observed in other research studies indicating increased occurrences of postprocedural adverse outcomes and MACE after PCI in women. For instance, in the meta-analysis conducted by Thandra et al which examined the PCI for left main coronary disease in women compared with men, women similarly demonstrated higher rates of MACE and MI with comparable rates of all-cause mortality and cardiac mortality.^42^ The study of 18,334 coronary artery disease patients undergoing PCI revealed that women had a higher unadjusted risk of all-cause mortality, cardiac mortality, or stroke compared to men. Even after adjusting for factors, women still faced greater risks of these outcomes, with the excess risk most significant in the first 30 days post-PCI.^43^ Huang et al conducted a study on sex disparity in the safety and efficacy of percutaneous transradial coronary intervention and transfemoral interventions. Fifteen studies involving 3,921,848 participants were included, and it was demonstrated that in both procedures, women were more prone to bleeding complications and MACE-related outcomes (death, MI, target vessel revascularization, or stroke), with lower sex differences in the transradial approach.^44^

Several studies reported varying results on post-PCI outcomes considering cardiac events by sex. In a study by Lee et al., this factor was not independently associated with adverse cardiac events following implantation of next-generation drug-eluting stents for long stenosis or chronic total occlusion lesions. Limited by small sample size and low events rate, findings had minimal sex association, and generalizability beyond the study’s population (mainly Asian population of Korean origin) remains uncertain.^45^ Additionally, Singh et al studied sex-based mortality differences after PCI over 25 years, finding improved 30-day mortality for both sexes in recent years. Long-term survival rates were similar between men and women, with no significant mortality differences after 1994 when adjusting for risk factors.^46^ Moreover, Chichareon et al^47^ conducted a prespecified subgroup analysis evaluating 16,000 patients in the GLOBAL LEADERS trial. After a follow-up for 1 year, it was demonstrated that women were more prone to bleeding and hemorrhagic stroke following PCI. However, other cardiovascular outcomes, such as all-cause mortality, MI, stent thrombosis, and repeat revascularization were comparable among men and women. The aforementioned contrary data could be due to the difference in the type of ACS (STEMI vs non-ST-elevation myocardial infarction) and the proportion of patients with stable versus unstable coronary syndrome.^47^

Repeat revascularization in all time frames showed no significant differences between men and women patients. In this regard, various findings have been documented in other studies. Kosmidou et al which assessed long-term outcomes following PCI, demonstrated a higher risk of target lesion revascularization (TLR) in women in a 5-year follow-up in addition to similar rates of cardiac death, and all-cause mortality between 2 groups.^48^ In another investigation, women exhibited comparable levels of TLR, yet notable heterogeneity was observed, with contemporary trials indicating elevated TLR rates.^42^

There are several explanations for the greater adverse events and mortality rates in women following the procedures than in men. Several studies have attributed the higher incidence of post-PCI complications in women to their older age and the increased incidence of associated disorders. This can be linked to the delayed manifestation and potential delays in the diagnosis of coronary artery disease. The delayed recognition could be due to the uncommon symptoms at the initiation of these conditions.^49^^,^^50^ Women exhibit a higher susceptibility to ACS and often present with specific lesion characteristics, such as ostial or shaft disease. These factors may contribute to worse outcomes, particularly considering that bifurcation disease occurs more frequently in this population.^42^ Moreover, women’s lower incomes may exacerbate sex disparities in revascularization outcomes, underscoring the need for further investigation and interventions to improve cardiovascular outcomes in the women’s investigation field.^51^ Moreover, due to the importance of lipid profiles in the prediction of cardiovascular accidents, differences in lipid parameters among men and women should be considered.^52^ Besides, plaque erosions and microvascular embolizations represent a significant pathological process that underlies the occurrence of ACS. These are more prominent in women patients, which could be the underlying cause of more adverse outcomes in women.^53^^,^^54^ For instance, in a recent cohort study conducted by Cenko et al, angiographic analysis demonstrated that women presenting with STEMI were more prone to experience suboptimal thrombosis in myocardial infarction blood flow (0-2), despite having minimal residual diameter stenosis (less than 25%). This observation persisted even after adjusting the baseline characteristics, including the duration from symptom onset to hospital arrival, indicating underlying sex differences in coronary physiology or response to PCI.^55^

Despite the well-established benefits of PCI in improving cardiovascular outcomes in the context of acute myocardial infarction, recognizing the worse outcomes for women after PCI necessitates increased vigilance in their care. This includes more aggressive treatment approaches, improved follow-up protocols, lower thresholds for diagnostic tests, and tailored interventions. This observed disparity may be indicative of underlying health inequities between men and women. Addressing these inequities may require a concerted effort to enhance attention to women's health issues and to develop treatment protocols, encompassing the selection of optimal medications, procedures, and post-PCI care plans that are specifically designed to meet their unique needs.^56^

### Study strengths and limitations

To the best of our knowledge, this article is one of the few systematic reviews and meta-analyses that examines the differences between men and women in terms of MACE outcomes after PCI. The notable findings have practical implications for designing personalized treatment plans for ACS patients. Our study is unique among previously published works because of the large number of reviewed articles and the analysis of MACE outcomes across 3 distinct time periods. Furthermore, this article represents the most current research on this topic, encompassing a comprehensive analysis of various outcomes. Despite its strengths, it is important to acknowledge certain limitations in our analysis. Firstly, our study lacked subgroup analyses due to multiple factors, including the inadequate distribution of studies based on scientifically validated cutoffs for subgroup analysis. Secondly, various factors may influence PCI outcomes, such as the number of occluded vessels, stent types, the baseline lipid profile of each patient, and diverse post-PCI therapeutic approaches. Thirdly, there may be variations in the duration of periods assessed for short-, mid-, and long-term outcomes across different articles. Moreover, we excluded patients with critical conditions, such as cardiogenic shock. These results could be different in these patients due to their state. Lastly, a notable limitation of our study lies in the inconsistent criteria used to define MACE components. Addressing this limitation represents a significant area of interest for future research. Additional efforts should be made in future studies to create more precise subgroups based on various factors influencing MACE outcomes and to acknowledge external influences that impact the analysis.

## Conclusions

ACS patients undergoing PCI encompass a significant portion of individuals admitted due to cardiovascular complications. The analysis from our meta-analysis suggests that women with ACS who undergo PCI have a higher incidence rate of MACE-related outcomes compared with men, in short-, mid-, and long-term.

### Data-sharing statement

The authors declare that the data supporting the findings presented in this study are accessible within both the main article and the supplementary materials provided.

### Ethical approval

This meta-analysis was performed in alignment with ethical guidelines pertaining to research involving human subjects. Given that the analysis aggregated data from previously published studies without engaging directly with participants, formal ethical approval was not necessary. Each of the studies included in this review had previously undergone ethical review by their respective Institutional Review Boards or ethics committees.

Perspectives **COMPETENCY IN PRACTICE-BASED LEARNING:** This systematic review and meta-analysis demonstrated that women diagnosed with acute coronary syndrome (ACS) undergoing percutaneous coronary intervention (PCI) face a higher risk of major adverse cardiovascular outcome (MACE)-related outcomes compared with men across various time frames.

**TRANSLATIONAL OUTLOOK:** This study highlights the significance of sex as a fundamental characteristic of patients and its role as a risk factor. It also emphasizes the potential for integrating sex as a variable in clinical practice to enhance cardiovascular risk stratification and management of patients following percutaneous coronary intervention (PCI).

## Funding support and author disclosures

This research did not receive any specific grant from funding agencies in the public, commercial, or not-for-profit sectors. The authors have reported that they have no relationships relevant to the contents of this paper to disclose.
