# Supplementary material for: Time Trends in Major Adverse Cardiovascular Events After Percutaneous Coronary Intervention: Meta-Analysis on Sex Differences
Source: JACC Adv. 2025 Jan 8;4(2):101526. doi: 10.1016/j.jacadv.2024.101526 (PMC11780094; doi:10.1016/j.jacadv.2024.101526)
Supplement: Supplemental Data [file mmc1.docx]

**Supplemental material**

| Database | Search terms | Result number |
| --- | --- | --- |
| PubMed | ("Gender"[Title/Abstract] OR "Sex"[Title/Abstract] OR "Women AND Men"[Title/Abstract] OR "Males AND Females"[Title/Abstract] OR "sex-specific"[Title/Abstract] OR "sex-disaggregated"[Title/Abstract]) AND ("stent*"[Title/Abstract] OR "catheterization*"[Title/Abstract] OR "catheter*"[Title/Abstract] OR "cannulation*"[Title/Abstract] OR "percutaneous coronary intervention*"[Title/Abstract] OR "percutaneous coronary revascularization*"[Title/Abstract] OR "revascularization percutaneous coronary"[Title/Abstract] OR "revascularizations percutaneous coronary"[Title/Abstract]) AND ("major adverse"[Title/Abstract] OR "cardiac event*"[Title/Abstract] OR "adverse"[Title/Abstract] OR "hospitalization*"[Title/Abstract] OR "rehospitalization*"[Title/Abstract] OR "re hospitalization*"[Title/Abstract] OR "readmission*"[Title/Abstract] OR "revascularization*"[Title/Abstract] OR "mi"[Title/Abstract] OR "myocardial infarction*"[Title/Abstract] OR "myocardial infarct*"[Title/Abstract] OR "heart attack*"[Title/Abstract] OR "stroke*"[Title/Abstract] OR "Mortality"[Title/Abstract] OR "death"[Title/Abstract] OR "End-Of-Life"[Title/Abstract] OR "cardiac accident*"[Title/Abstract] OR "cardiovascular accident*"[Title/Abstract]) AND ("difference*"[Title/Abstract] OR "impact"[Title/Abstract] OR "comparison*"[Title/Abstract] OR "comparing"[Title/Abstract] OR "compare"[Title/Abstract] OR "distinction"[Title/Abstract] OR "dissimilarity"[Title/Abstract]) AND ("random allocation*"[Title/Abstract] OR "random allocation*"[Title/Abstract] OR "double blind method*"[Title/Abstract] OR "double blind method*"[Title/Abstract] OR "single-blind-method"[Title/Abstract] OR "single-blind-method"[Title/Abstract] OR "singl*"[Title/Abstract] OR "doubl*"[Title/Abstract] OR "tripl*"[Title/Abstract] OR "blind*"[Title/Abstract] OR "mask*"[Title/Abstract] OR "random*"[Title/Abstract] OR "control*"[Title/Abstract] OR "controlled clinical trial*"[Title/Abstract] OR "comparative stud*"[Title/Abstract]) | 1238 |
| Embase | ('gender':ti,ab OR 'sex':ti,ab OR 'sex-specific':ti,ab OR 'sex-disaggregated':ti,ab OR 'women and men':ti,ab OR 'males and females':ti,ab) AND ('stent*':ti,ab OR 'catheterization*':ti,ab OR 'catheter*':ti,ab OR 'angioplast*':ti,ab OR 'cannulation*':ti,ab OR 'percutaneous coronary intervention*':ti,ab OR 'percutaneous coronary revascularization*':ti,ab OR 'revascularization percutaneous coronary':ti,ab OR 'revascularizations percutaneous coronary':ti,ab) AND ('major adverse':ti,ab OR 'cardiac event*':ti,ab OR 'adverse':ti,ab OR 'hospitalization*':ti,ab OR 'rehospitalization*':ti,ab OR 're-hospitalization*':ti,ab OR 'readmission*':ti,ab OR 'revascularization*':ti,ab OR 'mi':ti,ab OR 'myocardial infarction*':ti,ab OR 'myocardial infarct*':ti,ab OR 'heart attack*':ti,ab OR 'stroke*':ti,ab OR 'mortality':ti,ab OR 'death':ti,ab OR 'end-of-life':ti,ab OR 'cardiac accident*':ti,ab OR 'cardiovascular accident*':ti,ab) AND ('difference*':ti,ab OR 'impact':ti,ab OR 'comparison*':ti,ab OR 'comparing':ti,ab OR 'compare':ti,ab OR 'distinction':ti,ab OR 'dissimilarity':ti,ab) AND ('random allocation':ti,ab OR 'double-blind method':ti,ab OR 'single-blind method':ti,ab OR 'random':ti,ab OR 'control':ti,ab OR 'controlled clinical trial':ti,ab OR 'comparative study':ti,ab) AND [english]/lim AND 'article'/it | 1158 |
| Cochrane | ("Gender" OR "Sex" OR "Women AND Men" OR "Males AND Females" OR "sex-specific" OR "sex-disaggregated") AND ("stent" OR "catheterization" OR "catheter" OR "cannulation" OR "percutaneous coronary intervention" OR "percutaneous coronary revascularization" OR "revascularization percutaneous coronary" OR "revascularizations percutaneous coronary") AND ("major adverse" OR "cardiac event" OR "adverse" OR "hospitalization" OR "rehospitalization" OR "re hospitalization" OR "readmission" OR "revascularization" OR "mi" OR "myocardial infarction" OR "myocardial infarct" OR "heart attack" OR "stroke" OR "Mortality" OR "death" OR "End-Of-Life" OR "cardiac accident" OR "cardiovascular accident") AND ("difference" OR "impact" OR "comparison" OR "Comparing" OR "Compare" OR "distinction" OR "dissimilarity") AND ("random allocation" OR "double blind method" OR "single-blind-method" OR "single" OR "double" OR "triple" OR "blind" OR "mask" OR "random" OR "control" OR "controlled clinical trial" OR "comparative study") | 757 |
| ClinicalTrial.gov | Condition/disease: myocardial infarction  Other terms: Percutaneous Coronary Intervention | 429 |

*Supplementary Table 1. Search terms*

| Study characteristics | Male group | | | | | | | | | | Female group | | | | | | | | | |
| --- | --- | --- | --- | --- | --- | --- | --- | --- | --- | --- | --- | --- | --- | --- | --- | --- | --- | --- | --- | --- |
| Author/Year | Type of stent | Number of stents per person | Multivessel intervention  (%) | Unstable angina  (%) | STEMI  (%) | Non-STEMI  (%) | LM  (%) | LAD  (%) | RCA  (%) | LCX  (%) | Type of stent | Number of stents per person | Multivessel intervention  (%) | Unstable angina  (%) | STEMI  (%) | Non-STEMI  (%) | LM  (%) | LAD  (%) | RCA  (%) | LCX  (%) |
| Gimenez et al/2023 | N/A | N/A | 81 | N/A | N/A | N/A | N/A | 41 | 18 | 41 | N/A | N/A | 73/1 | N/A | N/A | N/A | N/A | 29 | 21 | 50 |
| Lee et al/2023 | SES | 1.38 | 20.8 | 27.9 | 38.4 | 33.6 | N/A | N/A | N/A | N/A | SES | 1.4 | 17.8 | 39.7 | 26.9 | 33.4 | N/A | N/A | N/A | N/A |
| Shin et al/2023 | DES | N/A | 50.25 | 30.8 | 11.4 | 15.9 | 2.05 | 60.7 | 36.2 | 25.9 | DES | N/A | 47.6 | 35.3 | 7.9 | 15 | 1.65 | 65.3 | 31.6 | 25.7 |
| Gabani et al/2022 | EES | 1.39 | 12.6 | N/A | 100 | N/A | 0.3 | 41.6 | 43.4 | 14.2 | EES | 1.37 | 12.2 | N/A | 100 | N/A | 0 | 47.7 | 40.1 | 11 |
| Madan et al/2022 | N/A | 1 | 42.8 | 29.5 | 22.4 | 29.1 | 2.5 | 51.6 | 35.3 | 27.4 | N/A | 1 | 40.5 | 34.7 | 19.8 | 28.9 | 2.1 | 51.6 | 36.1 | 25.2 |
| Yang et al/2022 | SES | 3 | 91.2 | 40.4 | N/A | N/A | 100 | N/A | N/A | N/A | SES | 2 | 90.3 | 50 | N/A | N/A | 100 | N/A | N/A | N/A |
| Eccleston et al/2021 | DES or BMS or both | N/A | N/A | N/A | N/A | N/A | N/A | N/A | N/A | N/A | DES or BMS or both | N/A | N/A | N/A | N/A | N/A | N/A | N/A | N/A | N/A |
| Verdoia et al/2021 | N/A | N/A | 36.3 | 23.25 | 46.2 | 30.6 | N/A | 12.5 | 13.1 | 9.95 | N/A | N/A | 29.75 | 31.9 | 26.9 | 41.2 | N/A | 12.0 | 10.5 | 9.25 |
| Vogel et al/2021 | DES | N/A | 65.05 | N/A | N/A | N/A | 5.1 | 54.8 | 35.1 | 34.2 | DES | N/A | 55.4 | N/A | N/A | N/A | 4.4 | 60.7 | 34.9 | 26.2 |
| Hara et al/2020 | First- generation PES | 5 | 95.8 | 27.5 | N/A | N/A | 37.3 | N/A | N/A | N/A | First- generation PES | 4 | 93.9 | 33.8 | N/A | N/A | 47 | N/A | N/A | N/A |
| Mehran et al/2020 | BMS or BioFreedom DCS | N/A | 65.25 | 14.75 | 16.1 | 83.8 | 3.9 | 53.5 | 38 | 31.7 | BMS or BioFreedom DCS | N/A | 56.4 | 16.35 | 15.35 | 84.6 | 3 | 57 | 37.9 | 27.5 |
| Gross et al/2019 | DES or BMS | N/A | 50.7 | N/A | 54.9 | 44.1 | 1.75 | 43.0 | 32.6 | 20.6 | DES or BMS | N/A | 41.0 | N/A | 54.8 | 45.2 | 0.95 | 41.9 | 38.3 | 17.7 |
| Venetsanos/2019 | N/A | N/A | N/A | N/A | N/A | N/A | N/A | N/A | N/A | N/A | N/A | N/A | N/A | N/A | N/A | N/A | N/A | N/A | N/A | N/A |
| Berry et al/2018 | DES or BMS | N/A | N/A | N/A | 15.3 | 16.7 | 0.74 | 38.8 | 23.4 | N/A | DES or BMS | N/A | N/A | N/A | 11.7 | 15 | 0.74 | 41.7 | 20.6 | N/A |
| Serruys et al/2018 | EES | 2.5 | N/A | N/A | N/A | N/A | 100 | N/A | N/A | N/A | EES | 2.3 | N/A | N/A | N/A | N/A | 100 | N/A | N/A | N/A |
| Shin et al/2018 | DES or BMS | N/A | 47.5 | N/A | 100 | 0 | N/A | 45.0 | 42.8 | 12.1 | DES or BMS | N/A | 55.4 | N/A | 100 | 0 | N/A | 48.0 | 43.7 | 8.2 |
| Gargiulo et al/2016 | BMS, ZES, PES, EES | N/A | 72.05 | 17.5 | 32.9 | 22.7 | N/A | N/A | N/A | N/A | BMS, ZES, PES, EES | N/A | 63.1 | 22.25 | 32.9 | 23.3 | N/A | N/A | N/A | N/A |
| Sia et al/2015 | EES | N/A | N/A | 15.55 | 34.6 | 49.8 | N/A | N/A | N/A | N/A | EES | N/A | N/A | 13.85 | 36.75 | 49.3 | N/A | N/A | N/A | N/A |
| Guagliumi et al/2014 | DES | 1.3 | 46.8 | N/A | 100 | N/A | N/A | 38.6 | 52.9 | 8.6 | DES | 1.4 | 40 | N/A | 100 | N/A | N/A | 50 | 42.9 | 7.1 |
| Regueiro et al/2014 | BMS or EES | N/A | 12.6 | N/A | 100 | N/A | N/A | N/A | N/A | N/A | BMS or EES | N/A | 12.3 | N/A | 100 | N/A | N/A | N/A | N/A | N/A |
| Tomey et al/2014 | DES | N/A | N/A | N/A | 100 | N/A | N/A | N/A | N/A | N/A | DES | N/A | N/A | N/A | 100 | N/A | N/A | N/A | N/A | N/A |
| Hansen et al/2013 | BMS or DES | 1.65 | 43.5 | N/A | 31 | N/A | N/A | 65.5 | 53 | 39.5 | BMS or DES | 1.65 | 39 | N/A | 33 | N/A | N/A | 64 | 54.5 | 30 |
| Meller et al/2013 | N/A | N/A | 48.8 | N/A | N/A | N/A | N/A | N/A | N/A | N/A | N/A | N/A | 48.9 | N/A | N/A | N/A | N/A | N/A | N/A | N/A |
| Tandjung et al/2013 | N/A | 2.01 | 24.95 | 21.8 | N/A | 29.0 | 2.75 | 42.2 | 28.8 | 23.4 | N/A | 2.03 | 22 | 27.45 | N/A | 25.9 | 2.2 | 39.5 | 36.0 | 21.4 |
| Ferrante et al/2012 | SES or BMS | 1.17 | 54.85 | N/A | N/A | N/A | N/A | N/A | N/A | N/A | SES or BMS | 1.07 | 49.7 | N/A | N/A | N/A | N/A | N/A | N/A | N/A |
| Kim et al/2012 | DES | 2.4 | 100 | 30.45 | N/A | N/A | N/A | 39.8 | N/A | N/A | DES | 2.25 | 100 | 38.8 | N/A | N/A | N/A | 38.0 | N/A | N/A |
| Mehilli et al/2012 | DES or BMS | N/A | 81.2 | N/A | N/A | N/A | 3.25 | 37.6 | 26.5 | 27.8 | DES or BMS | N/A | 75.9 | N/A | N/A | N/A | 2.25 | 41.8 | 28.3 | 26.5 |
| Tsujita et al/2010 | N/A | N/A | N/A | N/A | 100 | N/A | N/A | 40 | 45 | 14 | N/A | N/A | N/A | N/A | 100 | N/A | N/A | 36 | 50.5 | 12.5 |
| Lansky et al/2009 | EES | N/A | 37.6 | 21.3 | N/A | N/A | 0.3 | 39.2 | 30.5 | 29.8 | EES | N/A | 26.1 | 23.15 | N/A | N/A | 0 | 48.2 | 28.0 | 23.6 |
| Mehilli et al/2007 | DES or BMS | N/A | 76.15 | N/A | N/A | N/A | 2.15 | 40.7 | 27.2 | 25.0 | DES or BMS | N/A | 69 | N/A | N/A | N/A | 2.55 | 42.3 | 27.3 | 24.4 |
| Chako et al/2006 | N/A | N/A | 16.6 | N/A | N/A | N/A | N/A | N/A | N/A | N/A | N/A | N/A | 14.6 | N/A | N/A | N/A | N/A | N/A | N/A | N/A |
| Lansky et al/2005 | Multilink stent (Guidant Corp), | N/A | 48.9 | N/A | N/A | N/A | N/A | 37.6 | 43.9 | 18.4 | Multilink stent (Guidant Corp), | N/A | 48.4 | N/A | N/A | N/A | N/A | 34.5 | 50 | 15.3 |

*Supplementary Table2. Baseline characteristics of included studies (STEMI-ST-elevation myocardial infarction, LM-left main artery, LAD-left anterior descending artery, RCA-right coronary artery, LCX-left circumflex artery, DES-drug eluting stent, BMS-bare metal stent, SES-Sirolimus eluting stent, EES-Everolimus eluting stent, ZES-Zotarolimus eluting stent, PES-Paxlitaxel eluting stent, DCS-drug coated coronary stent)*

| Study characteristics | Male group | | | | | | | | | | | Female group | | | | | | | | | | |
| --- | --- | --- | --- | --- | --- | --- | --- | --- | --- | --- | --- | --- | --- | --- | --- | --- | --- | --- | --- | --- | --- | --- |
| Author/Year | Previous PCI | Previous CABG | Previous MI | Before intervention TIMI 0 | Before intervention TIMI 1 | Before intervention TIMI 2 | Before intervention TIMI 3 | After intervention TIMI 0 | After intervention TIMI 1 | After intervention TIMI 2 | After intervention TIMI 3 | Previous PCI | Previous CABG | Previous MI | Before intervention TIMI 0 | Before intervention TIMI 1 | Before intervention TIMI 2 | Before intervention TIMI 3 | After intervention TIMI 0 | After intervention TIMI 1 | After intervention TIMI 2 | After intervention TIMI 3 |
| Gimenez et al/2023 | 66.2 | N/A | 41.1 | N/A | N/A | N/A | N/A | N/A | N/A | N/A | N/A | 56.2 | N/A | 31.8 | N/A | N/A | N/A | N/A | N/A | N/A | N/A | N/A |
| Lee et al/2023 | 9.2 | 0.5 | 4.1 | N/A | N/A | N/A | N/A | N/A | N/A | N/A | N/A | 6.1 | 0.95 | 1.9 | N/A | N/A | N/A | N/A | N/A | N/A | N/A | N/A |
| Shin et al/2023 | N/A | N/A | 4.5 | N/A | N/A | N/A | N/A | N/A | N/A | N/A | N/A | N/A | N/A | 3.4 | N/A | N/A | N/A | N/A | N/A | N/A | N/A | N/A |
| Gabani et al/2022 | 4.2 | 0.8 | 5.5 | 58.4 | 8 | 13.6 | 20 | 1.5 | 0.8 | 4.1 | 93.5 | 3.5 | 0 | 4.7 | 63 | 6.3 | 11.8 | 18.9 | 2.8 | 0.8 | 3.2 | 93.3 |
| Madan et al/2022 | 24.7 | 8.1 | 15.5 | N/A | N/A | N/A | N/A | N/A | N/A | N/A | N/A | 18.8 | 9.6 | 10.9 | N/A | N/A | N/A | N/A | N/A | N/A | N/A | N/A |
| Yang et al/2022 | 12.7 | N/A | 5.3 | N/A | N/A | N/A | N/A | N/A | N/A | N/A | N/A | 12.5 | N/A | 1.4 | N/A | N/A | N/A | N/A | N/A | N/A | N/A | N/A |
| Eccleston et al/2021 | N/A | N/A | 28.8 | N/A | N/A | N/A | N/A | N/A | N/A | N/A | N/A | N/A | N/A | 17.2 | N/A | N/A | N/A | N/A | N/A | N/A | N/A | N/A |
| Verdoia et al/2021 | 11.5 | 3 | N/A | 27.8 | 11.7 | 15.85 | 44.7 | N/A | N/A | 0.15 | 99.85 | 7.5 | 1.9 | N/A | 17.25 | 7.25 | 16.05 | 59.45 | N/A | N/A | 0.4 | 99.6 |
| Vogel et al/2021 | 45.1 | 11.1 | 31.1 | N/A | N/A | N/A | N/A | N/A | N/A | N/A | N/A | 32.5 | 6.4 | 20.9 | N/A | N/A | N/A | N/A | N/A | N/A | N/A | N/A |
| Hara et al/2020 | N/A | N/A | 30.8 | N/A | N/A | N/A | N/A | N/A | N/A | N/A | N/A | N/A | N/A | 32.3 | N/A | N/A | N/A | N/A | N/A | N/A | N/A | N/A |
| Mehran et al/2020 | 23.9 | 11.4 | 22.1 | N/A | N/A | N/A | N/A | N/A | N/A | N/A | N/A | 17.8 | 5.95 | 16.8 | N/A | N/A | N/A | N/A | N/A | N/A | N/A | N/A |
| Gross et al/2019 | 14.8 | 3.65 | 12.4 | 38.8 | 13.35 | 23.9 | 23.95 | N/A | N/A | 2.75 | 97.25 | 9.85 | 1.8 | 6.85 | 40.7 | 12.55 | 23.85 | 22.9 | N/A | N/A | 3.4 | 96.6 |
| Venetsanos/2019 | 15.6 | 5.55 | 17.3 | N/A | N/A | N/A | N/A | N/A | N/A | N/A | N/A | 12.1 | 3 | 13.3 | N/A | N/A | N/A | N/A | N/A | N/A | N/A | N/A |
| Berry et al/2018 | 30.2 | 11.5 | 22.6 | N/A | N/A | N/A | N/A | N/A | N/A | N/A | N/A | 25.7 | 8.4 | 17.8 | N/A | N/A | N/A | N/A | N/A | N/A | N/A | N/A |
| Serruys et al/2018 | N/A | N/A | N/A | N/A | N/A | N/A | N/A | N/A | N/A | N/A | N/A | N/A | N/A | N/A | N/A | N/A | N/A | N/A | N/A | N/A | N/A | N/A |
| Shin et al/2018 | N/A | N/A | 3.4 | N/A | N/A | 2.4 | 1.15 | N/A | N/A | 4.85 | 91.15 | N/A | N/A | 0.55 | N/A | N/A | 2.95 | 0 | N/A | N/A | 10.35 | 86.2 |
| Gargiulo et al/2016 | 19.5 | N/A | 28.4 | N/A | N/A | N/A | N/A | N/A | N/A | N/A | N/A | 13.9 | N/A | 21.1 | N/A | N/A | N/A | N/A | N/A | N/A | N/A | N/A |
| Sia et al/2015 | 8.85 | 3.85 | 11.1 | N/A | N/A | N/A | N/A | N/A | N/A | N/A | N/A | 10.4 | 1.6 | 10.4 | N/A | N/A | N/A | N/A | N/A | N/A | N/A | N/A |
| Guagliumi et al/2014 | 1.4 | 1.4 | 2.9 | N/A | N/A | 27.1 | 11.4 | N/A | N/A | 7.1 | 92.9 | 1.4 | 0 | 0 | N/A | N/A | 35.7 | 7.1 | N/A | N/A | 4.3 | 95.7 |
| Regueiro et al/2014 | 4.2 | 0.8 | 5.5 | N/A | N/A | N/A | N/A | N/A | N/A | N/A | N/A | 3.3 | 0 | 4.45 | N/A | N/A | N/A | N/A | N/A | N/A | N/A | N/A |
| Tomey et al/2014 | 2.7 | N/A | 1.2 | N/A | N/A | N/A | N/A | N/A | N/A | N/A | N/A | 0.8 | N/A | 0 | N/A | N/A | N/A | N/A | N/A | N/A | N/A | N/A |
| Hansen et al/2013 | 13 | 3.5 | 12.5 | N/A | N/A | N/A | N/A | N/A | N/A | N/A | N/A | 9 | 2 | 10.5 | N/A | N/A | N/A | N/A | N/A | N/A | N/A | N/A |
| Meller et al/2013 | 13.3 | 2.6 | 16.6 | 61.8 | 7.5 | 10 | 20.8 | N/A | N/A | N/A | N/A | 6.3 | 1.6 | 7.7 | 58.7 | 7.4 | 9.3 | 24.6 | N/A | N/A | N/A | N/A |
| Tandjung et al/2013 | 21.4 | 11.8 | N/A | 5.5 | 3.85 | 7.15 | 83.5 | N/A | N/A | N/A | N/A | 18.8 | 7.6 | N/A | 6.05 | 4.15 | 5.15 | 84.65 | N/A | N/A | N/A | N/A |
| Ferrante et al/2012 | 6.4 | N/A | 9.55 | N/A | N/A | 11.5 | 18.95 | N/A | N/A | 4.6 | 93.6 | 2.2 | N/A | 1.7 | N/A | N/A | 15.6 | 27.9 | N/A | N/A | 3.4 | 94.3 |
| Kim et al/2012 | 28.3 | N/A | 37.2 | N/A | N/A | N/A | N/A | N/A | N/A | N/A | N/A | 24.6 | N/A | 34.4 | N/A | N/A | N/A | N/A | N/A | N/A | N/A | N/A |
| Mehilli et al/2012 | 34.8 | 12.2 | 21.6 | 19.35 | 6.45 | 23.65 | 50.55 | 1.1 | 0.65 | 4.45 | 93.8 | 24.8 | 5 | 16.2 | 18.3 | 7.75 | 18.55 | 55.4 | 1.25 | 1.75 | 3 | 94 |
| Tsujita et al/2010 | 14.6 | 3.35 | 13.9 | N/A | N/A | N/A | N/A | N/A | N/A | N/A | 86.5 | 9.1 | 3.2 | 8.55 | N/A | N/A | N/A | N/A | N/A | N/A | N/A | 87 |
| Lansky et al/2009 | N/A | N/A | 22.0 | N/A | N/A | N/A | N/A | N/A | N/A | N/A | N/A | N/A | N/A | 12.7 | N/A | N/A | N/A | N/A | N/A | N/A | N/A | N/A |
| Mehilli et al/2007 | N/A | 11.9 | 26.2 | 13.85 | 5.85 | 21.8 | 58.5 | 1.1 | 0.55 | 2.3 | 96.05 | N/A | 6 | 18.0 | 9.4 | 6.85 | 18.4 | 65.35 | 0.8 | 0.85 | 0.95 | 97.4 |
| Chako et al/2006 | 36 | 20.2 | 39.6 | N/A | N/A | N/A | N/A | N/A | N/A | N/A | N/A | 31.8 | 13 | 29.5 | N/A | N/A | N/A | N/A | N/A | N/A | N/A | N/A |
| Lansky et al/2005 | 12.7 | N/A | 15.7 | N/A | N/A | N/A | 20.9 | N/A | N/A | N/A | 95 | 7.1 | N/A | 8.4 | N/A | N/A | N/A | 25.3 | N/A | N/A | N/A | 97.1 |

*Supplementary Table3. Baseline characteristics of included studies (PCI-percutaneous coronary intervention, CABG-coronary artery bypass graft, MI-myocardial infarction, TIMI-thrombolysis in myocardial infarction)*

*Supplementary Table4. Risk of bias assessment of the included RCTs conducted using RoB2. Green color stands for low risk of bias, yellow color for some concerns, and red color for high risk of bias.*

|  | Duration of follow-up | Number of trials | RR [95% CI] | I^2% | P value for heterogeneity |
| --- | --- | --- | --- | --- | --- |
| Stent thrombosis | Short term  Mid term  Long term | 4  13  5 | 1.52[0.82; 2.82]  1.00[0.76; 1.32]  0.86[0.55; 1.34] | 0%  0%  0% | 0.42  0.74  0.48 |
| Major bleeding | Short term  Mid term  Long term | 3  7  3 | 3.05[1.81; 5.15]  1.88[1.63; 2.17]  1.12[0.89; 1.41] | 27%  9%  0% | 0.24  0.36  0.77 |

*Supplementary Table 5. Secondary outcomes in short-term, mid-term, and long-term duration of follow-up (RR-relative risk, CI-confidence interval)*

| Primary Outcomes | | | Fixed-effect model | | Random-effect model | |
| --- | --- | --- | --- | --- | --- | --- |
| Outcome | | Subgroup | RR (95% CI) | P-value | RR (95% CI) | P-value |
| All-cause mortality | | Short term | 2.72 [2.07; 3.58] | 0.11 | 2.51 [1.70; 3.71] | 0.11 |
|  |  | Mid term | 1.40 [1.25; 1.57] | <0.01 | 1.44 [1.21; 1.72] | <0.01 |
|  |  | Long term | 1.27 [1.16; 1.40] | 0.47 | 1.29 [1.17; 1.42] | 0.47 |
| Cardiovascular mortality | | Short term | 2.12 [0.79; 5.68] | 0.56 | 2.16 [0.80; 5.85] | 0.56 |
|  |  | Mid term | 1.22 [1.02; 1.46] | 0.03 | 1.29 [1.00; 1.66] | 0.03 |
|  |  | Long term | 1.26 [1.08; 1.48] | 0.82 | 1.30 [1.11; 1.52] | 0.82 |
| Myocardial infarction | | Short term | 1.16 [1.01; 1.34] | 0.01 | 1.33 [1.00; 1.77] | 0.01 |
|  |  | Mid term | 1.16 [1.04; 1.28] | 0.53 | 1.17 [1.06; 1.29] | 0.53 |
|  |  | Long term | 0.97 [0.84; 1.12] | <0.01 | 1.05 [0.83; 1.34] | <0.01 |
| Stroke | | Short term | 4.35 [1.35; 14.04] | 0.29 | 4.75 [0.83; 27.31] | 0.29 |
|  |  | Mid term | 1.49 [1.15; 1.93] | 0.67 | 1.47 [1.13; 1.92] | 0.67 |
|  |  | Long term | 1.09 [0.59; 2.01] | 0.56 | 1.19 [0.63; 2.26] | 0.56 |
| Need for revascularization | | Short term | 1.10 [0.85; 1.43] | 0.63 | 1.16 [0.89; 1.51] | 0.63 |
|  |  | Mid term | 1.12 [0.98; 1.28] | 0.12 | 1.13 [0.94; 1.35] | 0.12 |
|  |  | Long term | 0.87 [0.77; 0.98] | 0.19 | 0.89 [0.77; 1.04] | 0.19 |
| Secondary Outcomes | | | Fixed-effect model | | Random-effect model | |
| Outcome | Subgroup | | RR (95% CI) | P-value | RR (95% CI) | P-value |
| Stent thrombosis | Short term | | 1.35 [0.76; 2.40] | 0.42 | 1.52 [0.82; 2.82] | 0.42 |
|  | Mid term | | 0.89 [0.68; 1.17] | 0.74 | 1.00 [0.76; 1.32] | 0.74 |
|  | Long term | | 0.70 [0.46; 1.08] | 0.48 | 0.86 [0.55; 1.34] | 0.48 |
| Major bleeding | Short term | | 2.87 [2.12; 3.87] | 0.24 | 3.05 [1.81; 5.15] | 0.24 |
|  | Mid term | | 1.86 [1.63; 2.12] | 0.36 | 1.88 [1.63; 2.17] | 0.36 |
|  | Long term | | 1.08 [0.86; 1.37] | 0.77 | 1.12 [0.89; 1.41] | 0.77 |

*Supplementary Table6. Sensitivity analysis comparing fixed-effect versus random-effect models*

*(RR-relative risk, CI-confidence interval)*


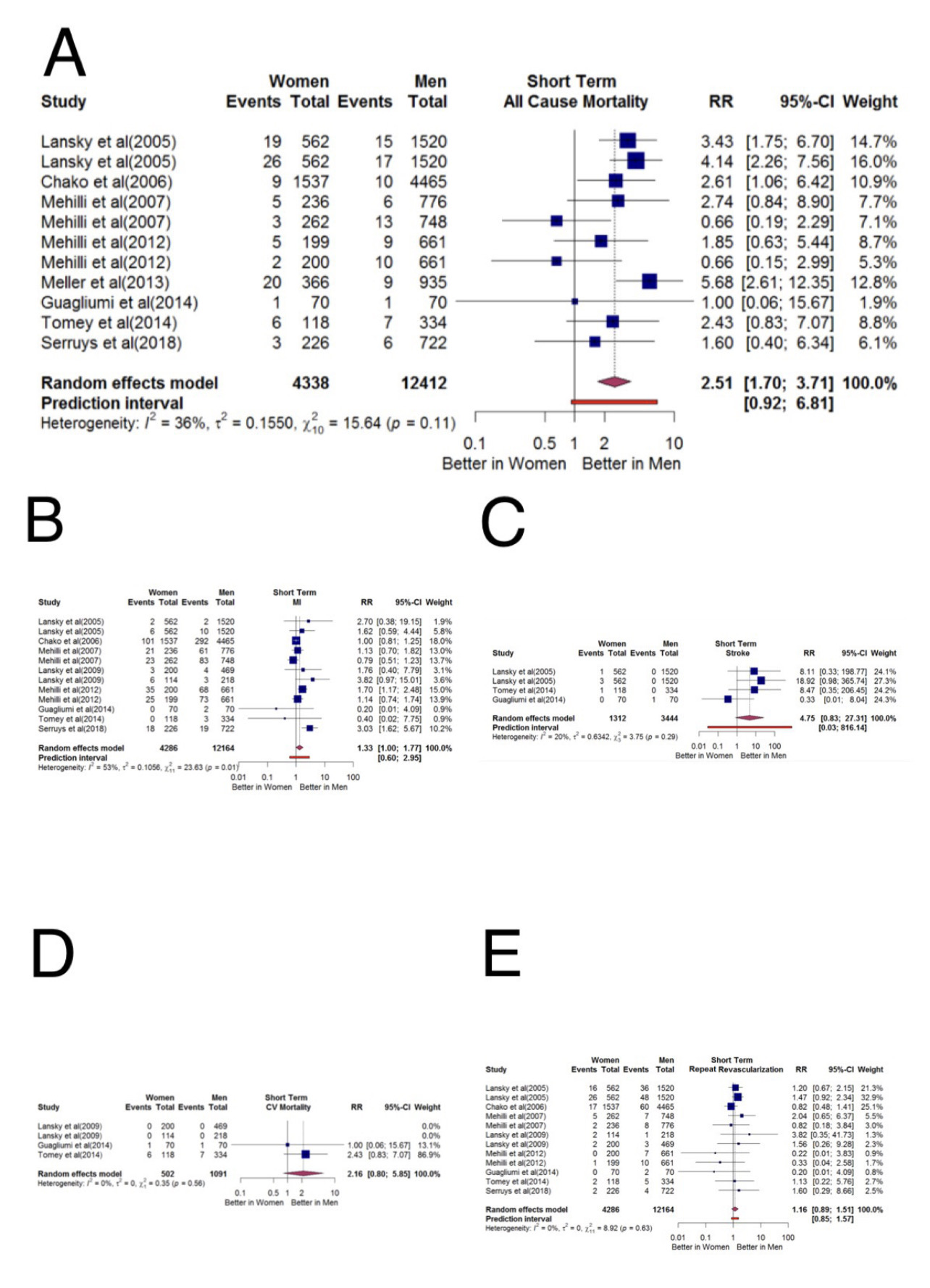


Supplementary Figure 1. (A) Risk ratio of short-term all-cause mortality (B) Risk ratio of short-term MI (C) Risk ratio of short-term stroke (D) Risk ratio of short-term CV mortality (E) Risk ratio of short-term repeat revascularization


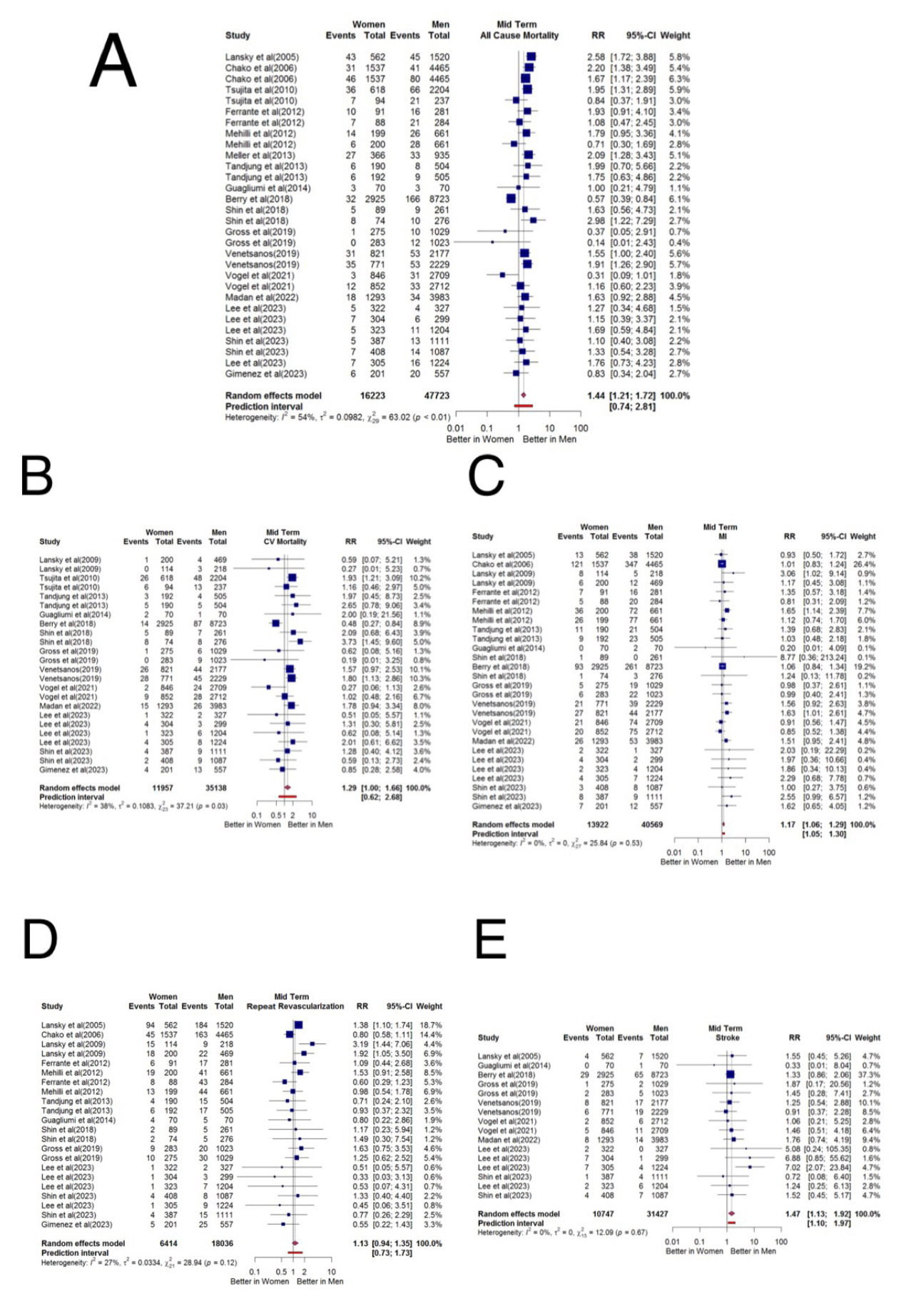


Supplementary Figure 2. (A) Risk ratio of mid-term all-cause mortality (B) Risk ratio of mid-term CV mortality (C) Risk ratio of mid-term MI (D) Risk ratio of mid-term need for revascularization (E) Risk ratio of mid-term stroke


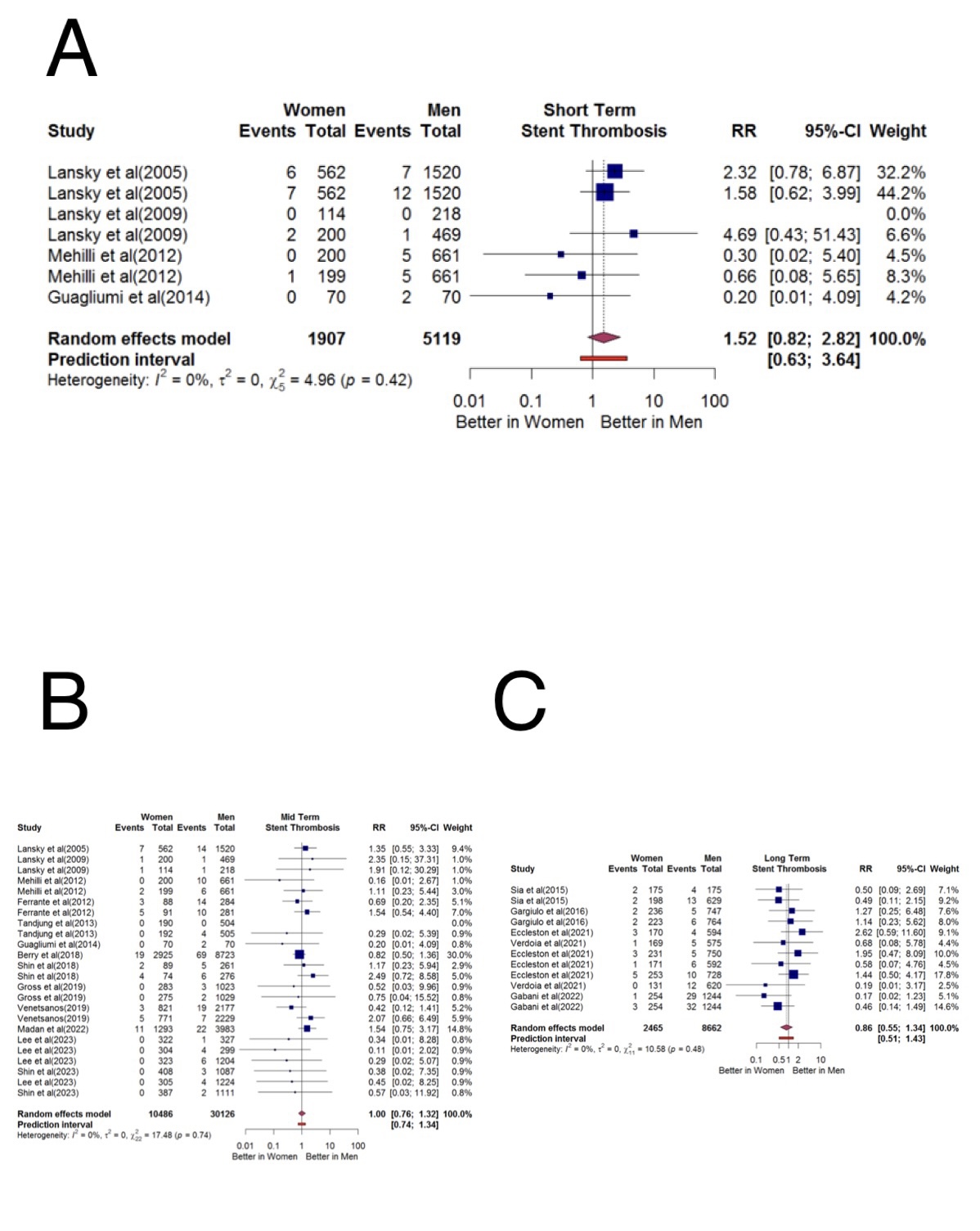


Supplementary Figure 3. (A) Risk ratio of short-term stent thrombosis (B) Risk ratio of mid-term stent thrombosis (C) Risk ratio of long-term stent thrombosis


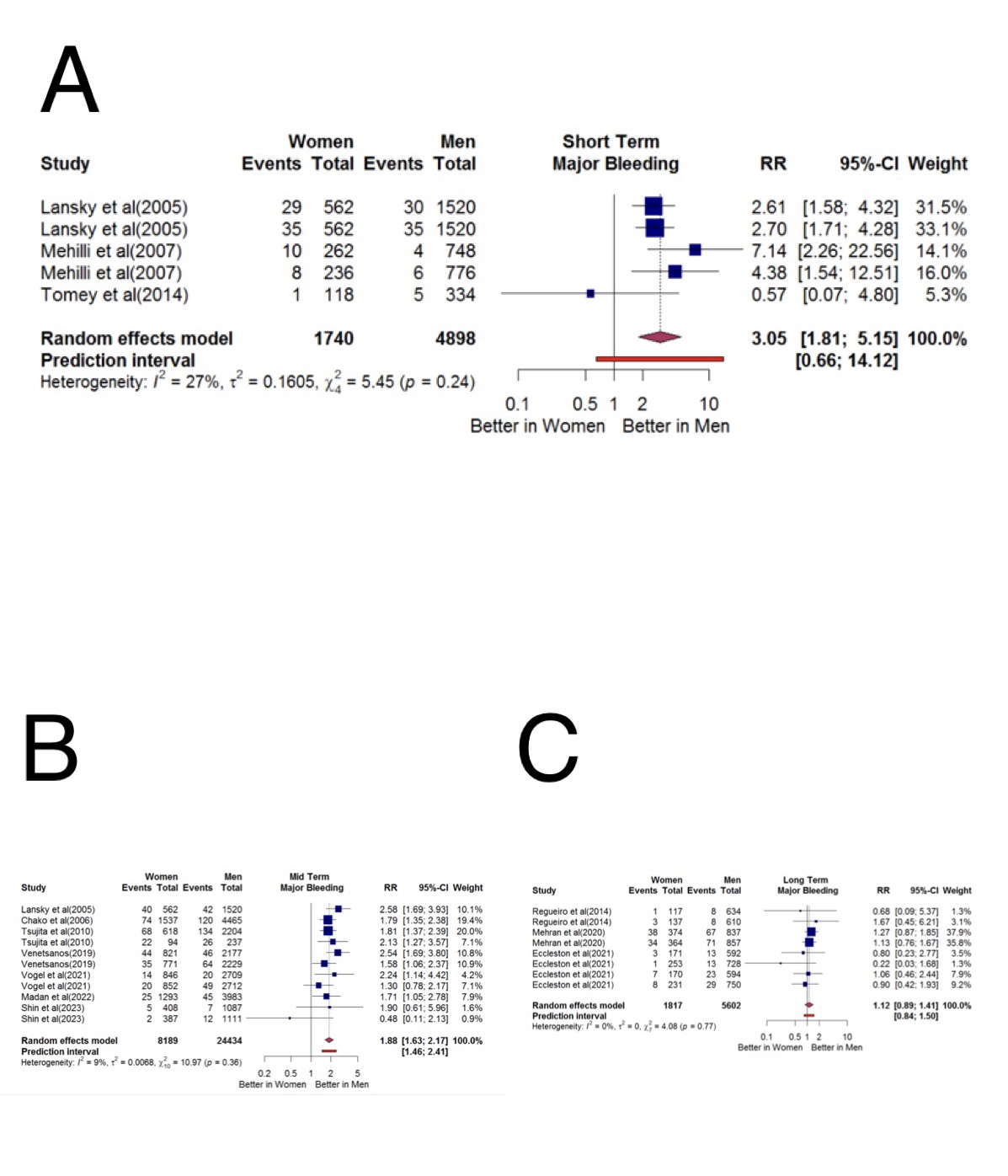


Supplementary Figure 4. (A) Risk ratio of short-term major bleeding (B) Risk ratio of mid-term major bleeding (C) Risk ratio of long-term major bleeding


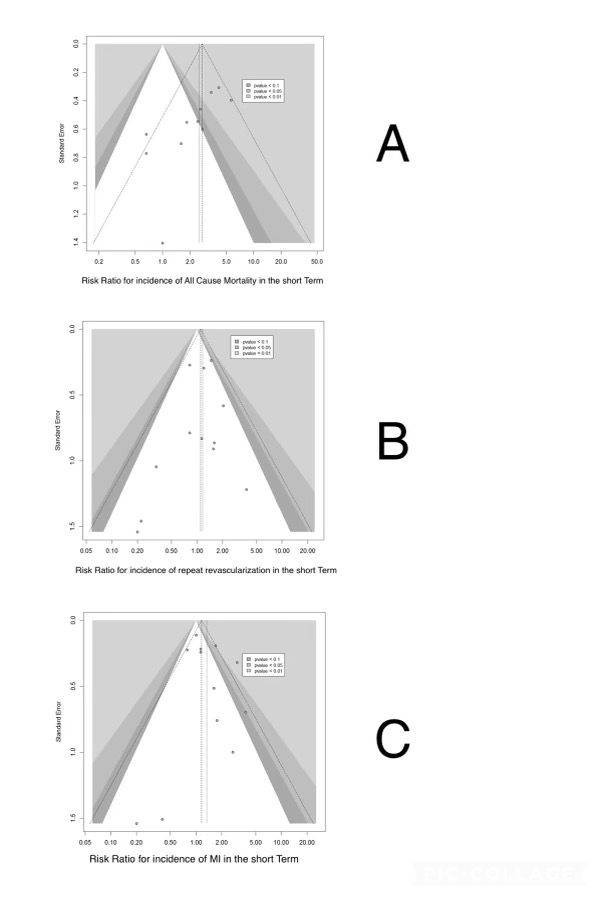


Supplementary Figure 5. (A) Funnel plot for all cause mortality in the short-term (B) Funnel plot for repeat revascularization in the short-term (C) Funnel plot for MI in the short-term


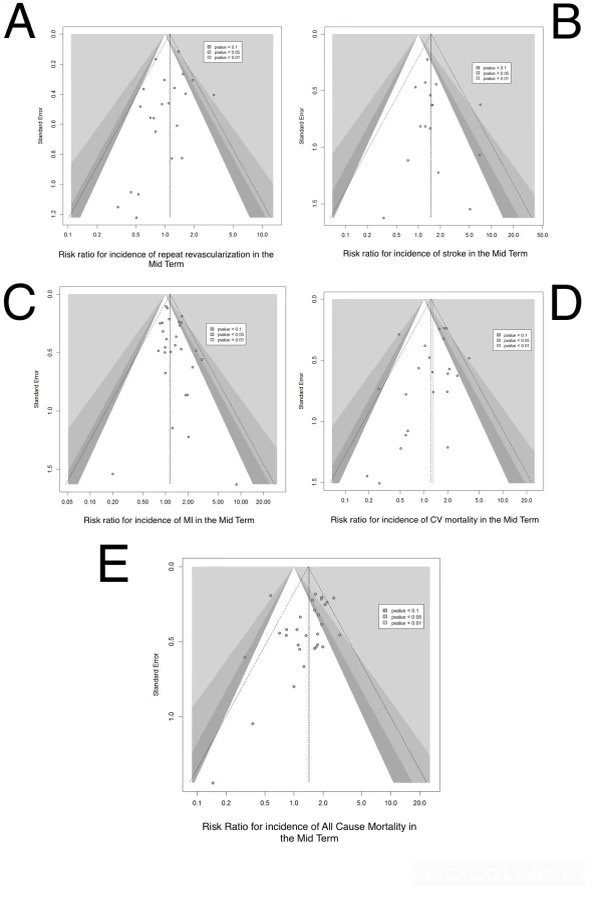


Supplementary Figure 6. (A) Funnel plot for repeat revascularization in the mid-term (B) Funnel plot for stroke in the mid-term (C) Funnel plot for MI in the mid-term (D) Funnel plot for CV mortality in the mid-term (E) Funnel plot for all cause mortality in the mid-term


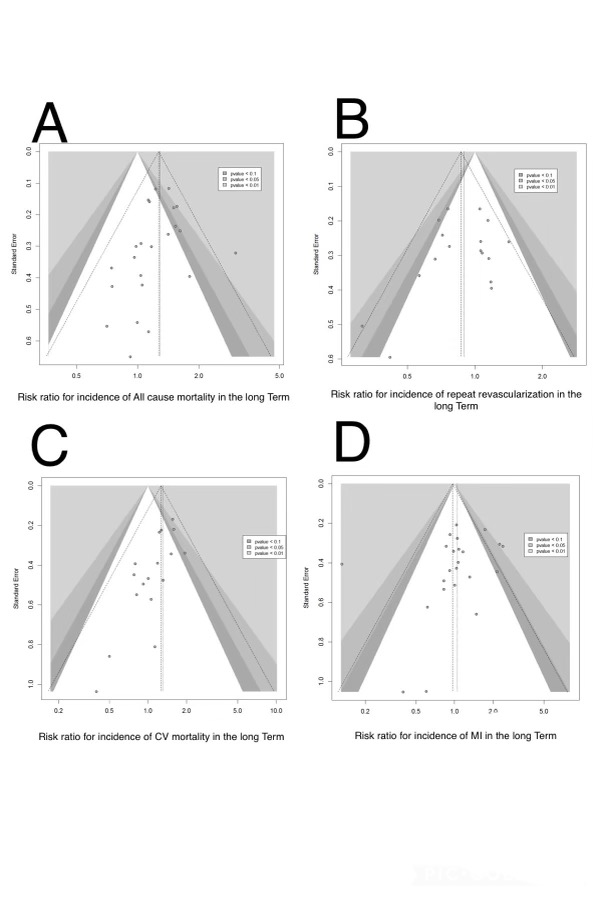


Supplementary Figure 7. (A) Funnel plot for all cause mortality in the long-term (B) Funnel plot for repeat revascularization in the long-term (C) Funnel plot for CV mortality in the long-term (D) Funnel plot for MI in the long-term


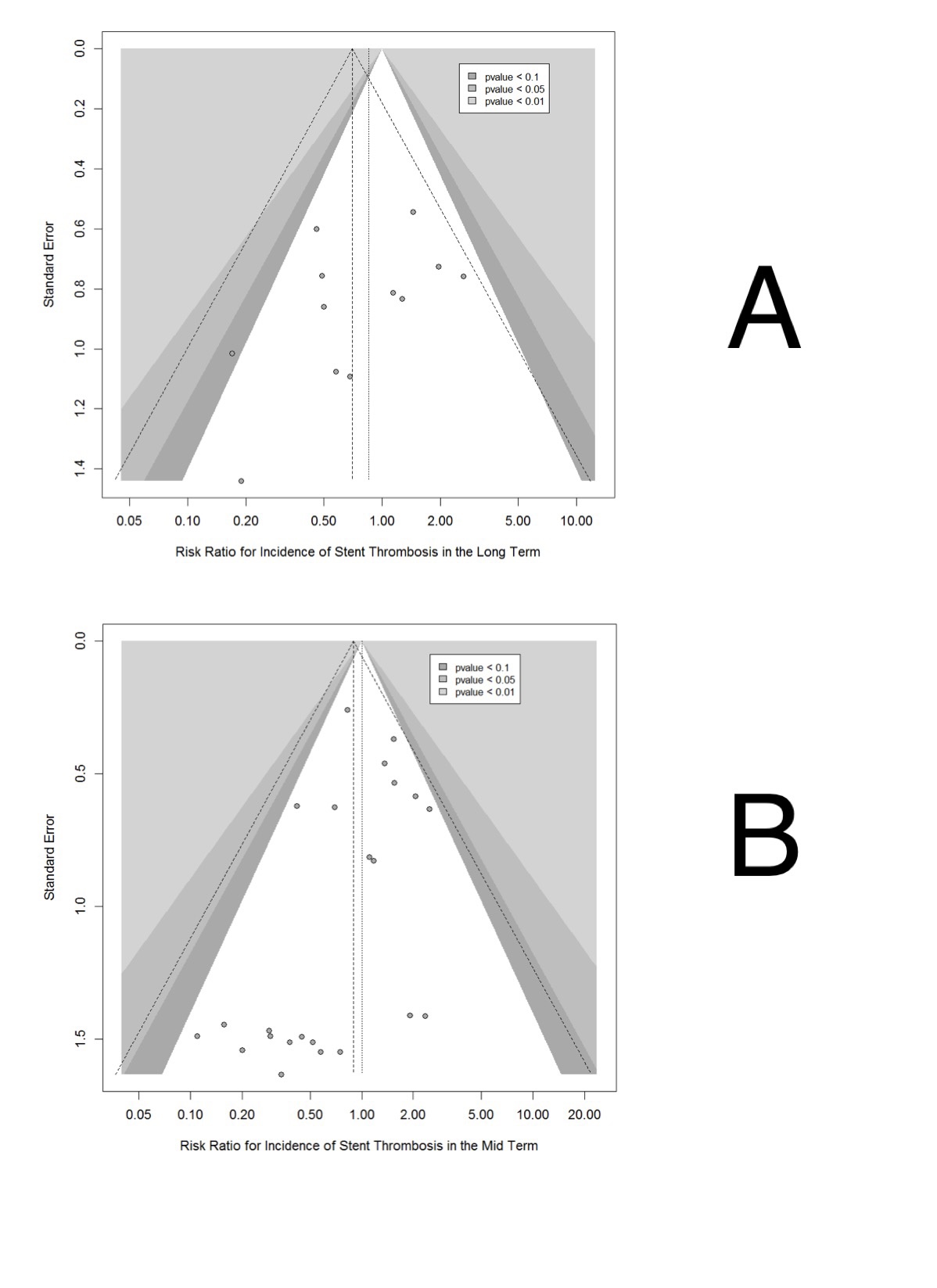


Supplementary Figure 8. (A) Funnel plot for stent thrombosis in the long-term (B) Funnel plot for stent thrombosis in the mid-term


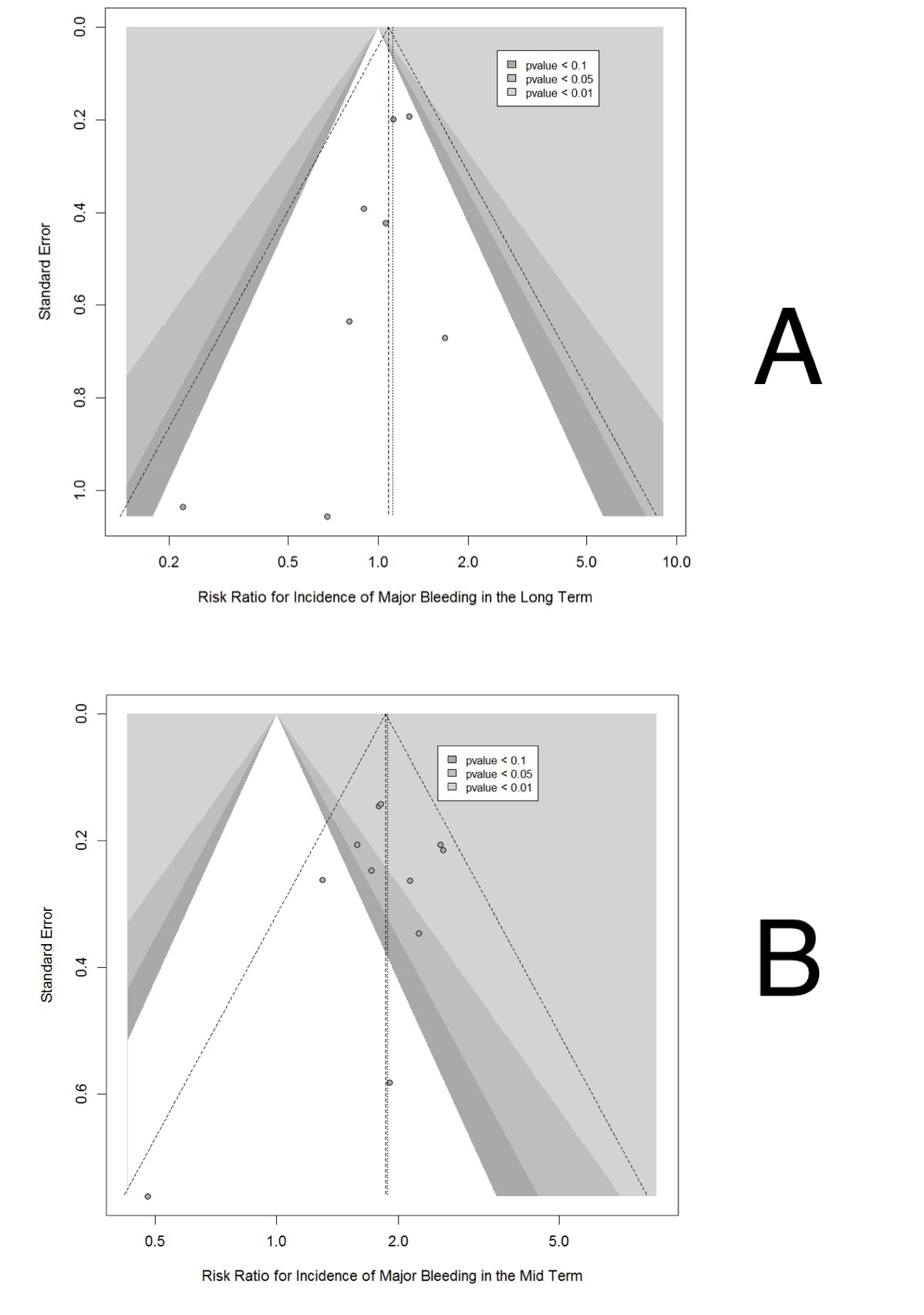


Supplementary Figure 9. (A) Funnel plot for major bleeding in the long-term (B) Funnel plot for major bleeding in the mid-term


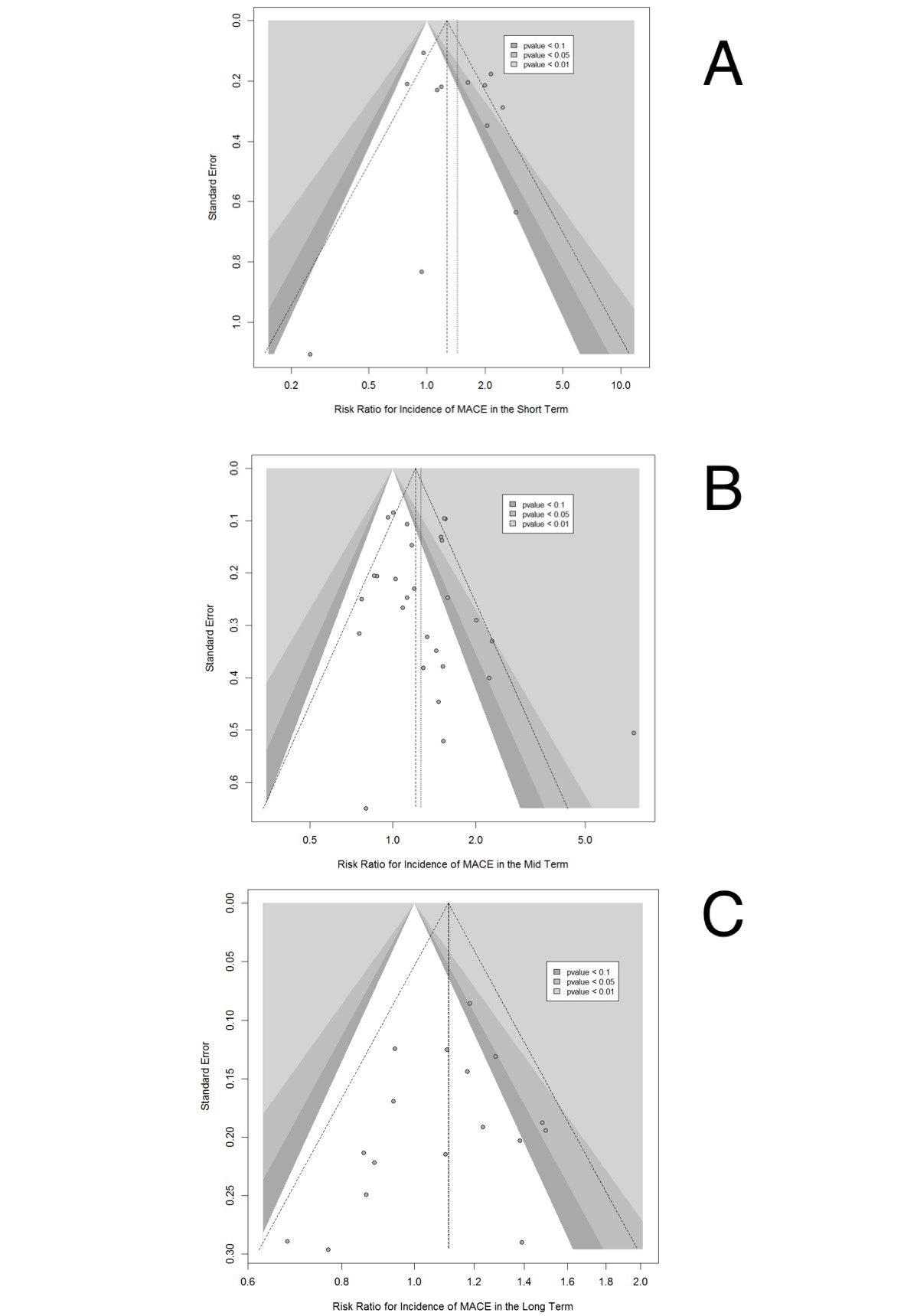


Supplementary Figure 10. (A) Funnel plot for MACE in the short-term (B) Funnel plot for MACE in the mid-term (C) Funnel plot for MACE in the long-term
